# Gestational-Age Clustering of Recurrent Placental Abruption: A Multicenter Retrospective Descriptive Study

**DOI:** 10.3390/jcm15145734

**Published:** 2026-07-22

**Authors:** Mako Nakamura, Kaori Iino, Takashi Ozaki, Makiko Sato, Yuki Osawa, Kanji Tanaka, Aisa Takeda, Sota Takahashi, Ryosuke Taniguchi, Hidetoshi Maruyama, Asami Ito, Yoshihito Yokoyama

**Affiliations:** 1 Department of Obstetrics and Gynecology, Graduate School of Medicine, Hirosaki University, Hirosaki 036-8562, Japan; 2 Perinatal Maternal and Child Center, Aomori Prefectural Central Hospital, Aomori 030-8553, Japan; 3 Department of Obstetrics and Gynecology, Aomori City Hospital, Aomori 030-0821, Japan; 4 Department of Obstetrics and Gynecology, Odate Municipal General Hospital, Odate 017-8550, Japan; 5 Department of Obstetrics and Gynecology, Hirosaki General Medical Center, Hirosaki 036-8545, Japan; 6 Department of Obstetrics and Gynecology, Mutsu General Hospital, Mutsu 035-8601, Japan; 7 Department of Obstetrics and Gynecology, Hachinohe City Hospital, Hachinohe 031-8555, Japan; 8 Department of Obstetrics and Gynecology, Tsugaru General Hospital, Goshogawara 037-0074, Japan; 9 Department of Obstetrics and Gynecology, Misawa City Hospital, Misawa 033-0022, Japan

**Keywords:** placental abruption, maternal vascular malperfusion, recurrent placental abruption, pregnancy surveillance, gestational age

## Abstract

**Background/Objectives**: Placental abruption has a high risk of recurrence, but the timing of recurrence relative to the previous episode remains unclear. This study described the gestational-age distribution of recurrent placental abruption and compared recurrent with first-time placental abruption in multiparous women. **Methods**: This retrospective multicenter descriptive study included 239 hospital-managed cases of placental abruption at nine perinatal centers in northern Japan from 2003 to 2024. Clinical characteristics and outcomes were compared between multiparous women with recurrent placental abruption (*n* = 10) and those with first-time placental abruption and no prior history of placental abruption (*n* = 134). Recurrence timing was assessed descriptively by comparing gestational age at onset between previous and current episodes. **Results**: Among 239 cases, 10 women had recurrent placental abruption. Maternal age was higher in the recurrent group, whereas BMI, smoking, assisted reproductive technology, and hypertensive disorders of pregnancy were similar between groups. Most clinical features and maternal and neonatal outcomes did not differ significantly, although birth weight was higher in the recurrent group. Among 11 recurrent events, 6 occurred within ±1 week (54.5%; exact 95% CI, 23.4–83.3%) and 8 within ±2 weeks (72.7%; exact 95% CI, 39.0–94.0%) of the previous gestational age. Available placental pathology in recurrent cases was reviewed descriptively only and was not interpreted as evidence of an association between maternal vascular malperfusion and recurrence. **Conclusions**: In this small exploratory cohort, recurrent placental abruption tended to occur within approximately ±1 to ±2 weeks of the gestational age of the previous episode, with 6 of 11 recurrent events occurring within ±1 week and 8 of 11 occurring within ±2 weeks. These exploratory findings may assist clinicians when individualizing surveillance strategies but should not be interpreted as supporting a specific surveillance protocol; confirmation in larger datasets is required.

## 1. Introduction

Placental abruption is a serious obstetric emergency characterized by the premature detachment of a normally implanted placenta before delivery [1,2]. This condition poses severe risks to both the mother and the fetus; in mothers, it may cause hemorrhagic shock, coagulopathy, and organ dysfunction due to massive bleeding, while in fetuses and neonates, it may lead to preterm birth, low birth weight, hypoxic injury, intrauterine fetal death, and neonatal death [1,2,3]. Although its incidence is relatively low, the condition has substantial clinical impact because of its rapid progression and its direct association with adverse maternal and neonatal outcomes [1,3,4].

Reported risk factors for placental abruption include hypertensive disorders of pregnancy, trauma, smoking, premature rupture of membranes, advanced maternal age, multiple pregnancy, and conception by assisted reproductive technology [1,2,3,4,5,6,7,8]. In clinical practice, however, placental abruption often develops unpredictably even in women without these typical risk factors, which limits pre-onset risk stratification [1,2,9]. In particular, a history of placental abruption is known to markedly increase the risk of recurrence in a subsequent pregnancy, serving as a strong risk marker even when other conventional risk factors are absent [1,2,4,10,11]. Familial risk has also recently been described, further supporting the clinical relevance of history-based surveillance that incorporates both obstetric and family history [9].

Despite its clinical importance, there is still insufficient evidence regarding the specific gestational age at which women with a history of placental abruption should commence high-risk management in a subsequent pregnancy [1,2,12,13]. Although major guidelines note the need for careful follow-up, there is a lack of concrete guidance on when surveillance should be intensified and how subsequent pregnancies should be monitored [1,12,13]. As a result, management in daily practice often relies on empirical judgment, and clinicians must individually balance the risk of recurrence against the burden of intervention, such as frequent visits and prolonged hospitalization [1,2,12,13]. In this context, information on whether recurrent placental abruption tends to occur near the gestational age of the previous episode may provide a clinically useful temporal reference, even if it cannot define a standardized management protocol. Therefore, clarifying practical timing for intensified monitoring after a previous abruption is clinically important, particularly for women who live far from emergency obstetric care.

Against this background, we retrospectively analyzed cases of placental abruption managed at nine perinatal care centers in northern Japan, with a specific focus on recurrent episodes. The aim of this study was to describe the gestational-age distribution of recurrent placental abruption relative to the previous episode and to compare the clinical characteristics and outcomes of recurrent cases with those of first-time cases in multiparous women. We also explored whether the gestational age at the previous episode could serve as a practical temporal reference for individualized surveillance planning in subsequent pregnancies, while recognizing the descriptive and exploratory nature of the study.

## 2. Materials and Methods

### 2.1. Study Design and Population

We conducted a retrospective multicenter descriptive study of placental abruption cases managed at nine perinatal care centers in northern Japan between January 2003 and December 2024. This study was designed to describe the gestational-age distribution of recurrent placental abruption relative to the previous episode and was not intended to estimate the population-level recurrence risk of placental abruption or to identify independent predictors of recurrence. The study was approved by the Institutional Review Board of Hirosaki University Graduate School of Medicine (No. 2022-119). The requirement for informed consent was waived due to the retrospective nature of the study, and an opt-out approach was used to allow eligible women to decline participation.

### 2.2. Data Collection and Definitions

A total of 239 women diagnosed with placental abruption were identified from medical records. Placental abruption was diagnosed primarily on clinical grounds, based on a combination of clinical presentation suggestive of placental abruption (e.g., genital bleeding, abdominal pain, uterine tenderness, abnormal fetal heart rate tracing), ultrasonographic findings suggestive of placental abruption when present, and placental findings at delivery, including retroplacental hematoma or adherent clot on the delivered placenta. Histopathological findings were used as supportive information when available. However, because this was a long-term retrospective multicenter study, histopathological examination was not performed or available in all cases, and pathology results could not be comprehensively confirmed across institutions. Therefore, diagnostic ascertainment was based primarily on clinical diagnosis and documentation at each participating institution and was not centrally adjudicated.

Gestational age was determined according to routine obstetric practice at each participating institution, based primarily on the last menstrual period and confirmed or revised by ultrasonographic findings when available. For pregnancies conceived by assisted reproductive technology, gestational age was calculated based on the date of oocyte retrieval, embryo transfer, or insemination, as appropriate. Because information on the exact basis for gestational-age determination was not systematically collected in this retrospective study, we could not reliably determine the proportion of cases in which gestational age was confirmed by ultrasonography versus based primarily on the last menstrual period. Gestational age at onset of placental abruption was defined as the gestational age at the first maternal symptoms, abnormal fetal heart rate tracing, ultrasonographic findings, or clinical findings that led to the diagnosis, as documented in the medical record.

To specifically investigate the characteristics of recurrence, we categorized multiparous women into two groups: (1) the recurrent placental abruption group (*n* = 10), consisting of women with placental abruption in the index pregnancy and a history of placental abruption in a previous pregnancy, and (2) the first-time placental abruption group (*n* = 134), consisting of multiparous women experiencing their first episode of placental abruption in the index pregnancy and having no prior history of placental abruption. Nulliparous women were excluded from this comparative analysis. For the analysis of recurrence timing, gestational ages at onset in both the previous and index pregnancies were reviewed among women in the recurrent group. Because one woman had two previous pregnancies complicated by placental abruption, 11 recurrent events were identified among 10 women.

### 2.3. Clinical Outcomes and Pathological Evaluation

Maternal and neonatal characteristics were extracted, including maternal age, body mass index (BMI), presence of hypertensive disorders of pregnancy (HDP), gestational age at delivery, and neonatal birth weight. Documented uterine conditions, including uterine leiomyomas and adenomyosis, were also extracted from medical records when available; however, systematic imaging review for these conditions was not performed. Clinical presentation, ultrasonographic findings, abnormal fetal heart rate tracing, pregnancy course, mode of delivery, maternal outcomes, and neonatal outcomes were also collected from medical records. Furthermore, we examined the gestational age at onset for both the current and previous pregnancies in the recurrent group to determine the temporal distribution of recurrence.

Placental pathological findings were reviewed from the original pathology reports and, when available, from archived histological slides. Findings suggestive of maternal vascular malperfusion (MVM) were described according to the Amsterdam Placental Workshop Group Consensus Statement when such information was available. However, because this study covered a long period from 2003 to 2024, during which the concept and standardized reporting of MVM became more widely recognized, the presence or absence of MVM-related lesions was not systematically documented in many historical pathology reports. In addition, archived placental slides from the first-time placental abruption group were not uniformly available for direct review. Therefore, pathological data in the first-time placental abruption group were not sufficiently complete or consistent to allow a formal pathological comparison with the recurrent group. For this reason, MVM-related findings were presented only as exploratory observations in recurrent cases for which pathological reports and/or archived histological slides were available, rather than as evidence of a pathological difference between recurrent and first-time placental abruption. Centralized blinded pathological review of all placental slides was not feasible in the present retrospective multicenter study.

### 2.4. Statistical Analysis

Continuous variables are expressed as medians with interquartile ranges (IQR) and were compared using the Mann–Whitney U test. Categorical variables are presented as numbers and percentages and were analyzed using Fisher’s exact test. Parity was compared using the Fisher–Freeman–Halton exact test. The temporal relationship between the gestational ages at previous and subsequent placental abruption episodes was examined descriptively. Because of the small number of recurrent cases, no multivariable adjustment, matching procedure, or regression-based modeling was performed. The proportions of recurrent events occurring within ±1 week and ±2 weeks of the gestational age at the previous episode were calculated descriptively, and exact 95% confidence intervals were used to indicate the imprecision of these estimates. Missing data were not imputed, and analyses were performed using available data for each variable. All statistical analyses were performed using R statistical software (version 4.2.2 or later). A *p*-value of <0.05 was considered statistically significant. All analyses were considered exploratory.

### 2.5. Ethical Considerations

This retrospective observational study was approved by the Ethics Committee of Hirosaki University Graduate School of Medicine as the lead institution (approval number: 2022-119; approval date: 17 February 2023). Depending on the ethical review system adopted at each participating institution, some institutions relied on the approval obtained from the lead institution and completed the required local institutional authorization or administrative procedures, whereas others underwent individual local ethics committee or IRB review. All required ethical review, approval, and institutional authorization procedures were completed at each participating institution according to its local policy. Given the retrospective nature of the study, informed consent was waived or addressed by an opt-out procedure in accordance with the ethical policies of each institution.

## 3. Results

### 3.1. Study Population and Baseline Characteristics

During the study period, 239 cases of placental abruption were identified. The baseline characteristics of the overall cohort are shown in Table 1. The median maternal age was 33.0 years, and the median pre-pregnancy BMI was 21.0 kg/m^2^. Of the total cohort, 4.2% (10/239) were identified as recurrent placental abruption cases, while 56.1% (134/239) were multiparous women experiencing their first placental abruption episode. Because this study included hospital-managed placental abruption cases and did not include all deliveries at the participating centers as a denominator, the observed recurrence proportion should not be interpreted as a population-level recurrence estimate. In addition, selection bias related to this hospital-managed cohort may have influenced both the observed recurrence proportion and recurrence-timing distribution.

### 3.2. Comparison Between Recurrent and First-Time Placental Abruption

Table 2 compares the maternal characteristics of the recurrent placental abruption group (*n* = 10) and the first-time placental abruption group (*n* = 134). Maternal age was significantly higher in the recurrent placental abruption group than in the first-time placental abruption group (35.5 [34.0–40.5] vs. 34.0 [30.0–37.0] years, *p* = 0.039). However, the absolute difference was modest and the interquartile ranges overlapped; therefore, this finding should not be overinterpreted as a clinically actionable difference. No significant differences were observed in BMI, smoking status, conception by assisted reproductive technology, obstetric history, or hypertensive disorders of pregnancy. Most clinical findings and maternal and neonatal outcomes were also comparable between groups (Table 3). Birth weight was significantly higher in the recurrent group (2600 [2287–2888] vs. 2146 [1543–2560] g, *p* = 0.011), whereas gestational age at diagnosis and delivery did not differ significantly. Given the small number of recurrent cases, these between-group comparisons were considered exploratory, and the absence of statistically significant differences should be interpreted cautiously.

### 3.3. Timing of Recurrence

**Figure 1:**
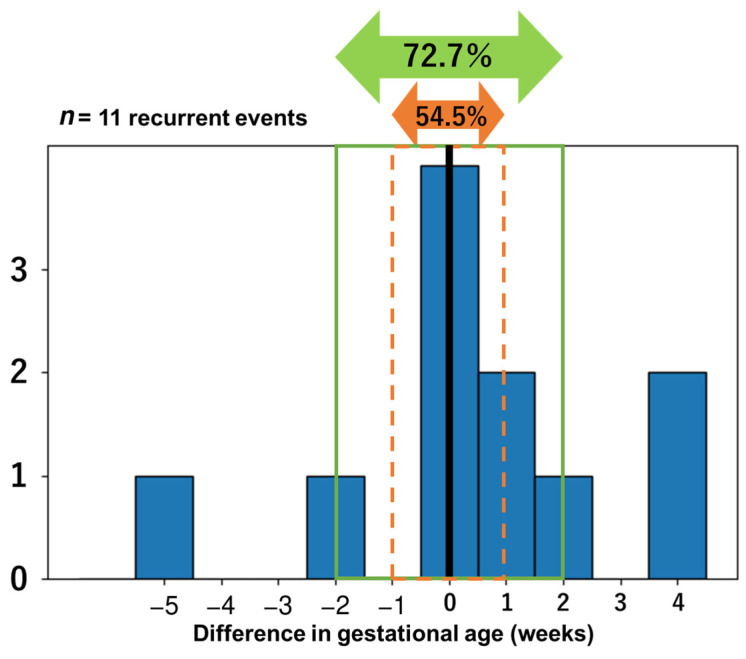
Timing of recurrent placental abruption relative to the previous episode. Differences in gestational age at placental abruption onset (current pregnancy minus previous pregnancy) are shown for 11 recurrent events in 10 pregnancies. One woman had two prior placental abruptions and therefore contributed two recurrent events. Six of 11 events occurred within ±1 week (54.5%; exact 95% CI, 23.4–83.3%), and 8 of 11 events occurred within ±2 weeks (72.7%; exact 95% CI, 39.0–94.0%) of the gestational age at the previous episode. The black vertical line indicates no difference in gestational age. The orange dashed lines delimit the ±1-week window, and the green outline delimits the ±2-week window; the orange and green double-headed arrows indicate the corresponding proportions of events within these windows.

The relationship between gestational age (GA) at onset in the previous and that in the current pregnancies was analyzed for 11 recurrent events among 10 women (Figure 1). Recurrent events tended to occur close to the gestational age at the previous episode. The median difference in GA between the previous and subsequent episodes was 0.0 weeks (IQR: −1.0 to +1.4 weeks). Specifically, 54.5% (6/11) of recurrences occurred within ±1 week of the GA at the previous episode (exact 95% confidence interval [CI], 23.4–83.3%), and 72.7% (8/11) occurred within ±2 weeks of the GA at the previous episode (exact 95% CI, 39.0–94.0%). Conversely, 3 of 11 recurrent events (27.3%) occurred outside the ±2-week window. Among these 3 events, 2 occurred 4 weeks later than the previous episode and recurred at term, at 37 weeks of gestation. The remaining event occurred 5 weeks earlier than the previous episode, recurred at 34 weeks of gestation, and resulted in neonatal death; in this case, the patient lived far from the hospital. These estimates were descriptive and showed wide confidence intervals because of the small number of recurrent events.

### 3.4. Placental Pathology in Recurrent Cases

**Figure 2:**
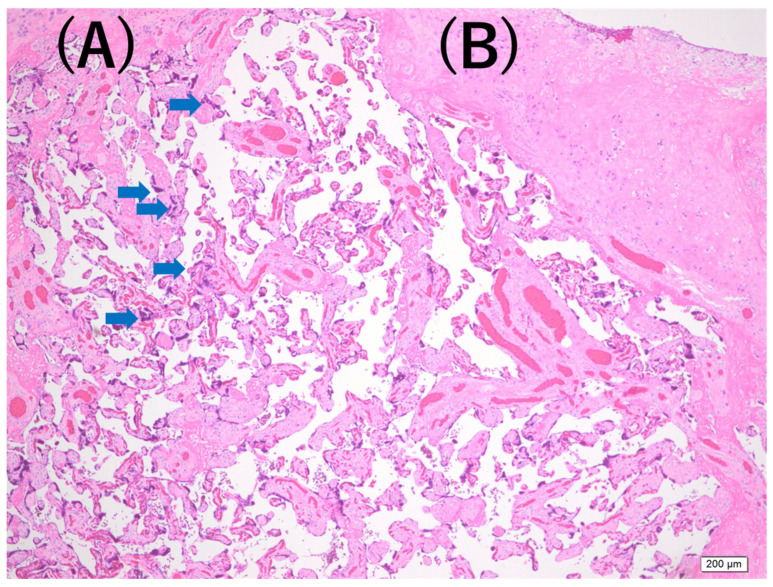
Representative placental pathology findings in the previous pregnancy of a recurrent placental abruption case. Findings suggestive of maternal vascular malperfusion in the placenta from the previous pregnancy are shown. (**A**) Increased syncytial knots (arrows) on hematoxylin and eosin staining. (**B**) Placental infarction on hematoxylin and eosin staining. Scale bar = 200 μm. These images are purely illustrative and do not support an association between MVM and recurrent placental abruption.

Available placental pathology reports and/or archived histological slides from recurrent cases were reviewed descriptively. Some available specimens showed findings suggestive of maternal vascular malperfusion (MVM), including extensive villous infarction, decidual vasculopathy, and increased syncytial knots (Figure 2). However, pathological assessment was not standardized across institutions or years, and centralized blinded pathological review was not performed, including within the recurrent cases. Therefore, the apparent frequency of MVM-related findings could not be regarded as a reliable estimate and was not used for quantitative interpretation.

In addition, comparable pathological data were not systematically available for the first-time placental abruption group. Therefore, the available MVM-related findings were summarized only as descriptive observations in recurrent cases and should not be interpreted as evidence that MVM is associated with recurrence, as evidence of a pathological difference between recurrent and first-time placental abruption, or as direct evidence of a causal mechanism of recurrence.

## 4. Discussion

This retrospective multicenter descriptive study included 239 cases of placental abruption from nine institutions in northern Japan and compared recurrent cases in multiparous women (*n* = 10) with multiparous first-time cases (*n* = 134), while also examining the distribution of recurrence timing in recurrent cases. The principal finding of this study was that recurrent events tended to occur within approximately ±1 to ±2 weeks of the gestational age at onset of the previous episode. Approximately half of recurrent events occurred within ±1 week of the gestational age at the previous episode, and approximately three-quarters occurred within ±2 weeks. Because the main recurrence-timing analysis was based on only 10 women and 11 recurrent events, and because the exact 95% confidence intervals were wide, these proportions should be interpreted as descriptive and hypothesis-generating rather than as precise estimates. Accordingly, the observed temporal clustering should be considered unstable, and both the timing pattern and the estimated proportions may change substantially in larger cohorts; confirmation in substantially larger multicenter studies is required. These findings suggest that the gestational age at the previous episode may serve as a practical temporal reference when planning individualized surveillance in a subsequent pregnancy among women with a history of placental abruption. Importantly, because recurrent placental abruption is rare and the recurrent group in this study was small, the present study was statistically underpowered to detect certain associations between recurrence and specific clinical characteristics, pathological findings, management factors, or maternal and neonatal outcomes.

Although known risk factors for placental abruption include hypertensive disorders of pregnancy, chronic hypertension, smoking, premature rupture of membranes, trauma, and conception by assisted reproductive technology, onset can also occur in women without these risk factors, limiting the ability to predict placental abruption in advance [1,2,3,9]. In the present study, maternal age was higher in the recurrent group, whereas the frequency of advanced maternal age itself was not significantly different. Likewise, no clear between-group differences were observed in the frequencies of previously reported clinical risk factors, including smoking, conception by assisted reproductive technology, previous cesarean delivery, previous preterm birth, previous hypertensive disorders of pregnancy, chronic hypertension, preexisting diabetes mellitus, hypertensive disorders of pregnancy in the index pregnancy, and fetal growth restriction. However, this analysis was not designed to identify independent risk factors for recurrence, and the small number of recurrent cases limited statistical power. In particular, the study was underpowered to detect potentially meaningful associations between recurrent placental abruption and infrequent clinical factors such as smoking, assisted reproductive technology, low-dose aspirin use, hypertensive disorders of pregnancy, fetal growth restriction, and severe neonatal outcomes. Therefore, the absence of between-group differences should not be interpreted as evidence that these factors are unrelated to recurrence. Rather, these comparisons should be viewed as descriptive clinical context for the recurrent cases and as inconclusive with respect to the presence or absence of specific associations.

The fact that recurrence could not be adequately explained by conventional clinical factors alone suggests that placental abruption may involve underlying pathophysiology that is not readily captured by routine clinical indicators. Recent work has suggested that both inflammatory pathways related to infection and chronic ischemic pathways related to impaired uteroplacental circulation may contribute to the pathogenesis of placental abruption [1,14,15,16,17,18]. This background supports the biological plausibility of placental vascular pathology in placental abruption in general, but the present study was not able to determine whether MVM is specifically associated with recurrent placental abruption. These findings further emphasize that women with a previous placental abruption should be informed early in pregnancy about recurrence risk and warning symptoms, and that clinicians should proactively plan an escalation of monitoring before the gestational age at which the previous episode occurred.

In the present study, available pathology reports and/or archived histological slides from recurrent cases included findings suggestive of MVM. However, these findings should be interpreted with particular caution. Standardized pathological data were not available for the first-time placental abruption group, and centralized blinded pathological review was not performed, including within the recurrent cases. Therefore, the apparent frequency of MVM-related findings in recurrent cases cannot be regarded as a reliable estimate and should not be used to infer that MVM is associated with recurrent placental abruption.

The present study covered a long period from 2003 to 2024, during which the concept and standardized reporting of MVM became more widely recognized. Consequently, many historical pathology reports did not systematically describe the presence or absence of MVM-related lesions, and archived placental slides were not uniformly available for direct review, particularly in the first-time placental abruption group. In addition, because diagnostic consistency of MVM-related findings could not be verified even within the recurrent cases, the apparent frequency of these findings may have been influenced by non-standardized pathological assessment, differences in reporting practices across institutions and years, and diagnostic heterogeneity. For these reasons, we intentionally refrained from performing a formal pathological comparison between recurrent and first-time cases, because such a comparison could have introduced substantial misclassification bias.

Accordingly, the pathological findings in this study should be regarded only as illustrative contextual observations based on available recurrent-case specimens. They should not be interpreted as evidence of a pathological difference between recurrent and first-time placental abruption, as evidence that MVM is associated with recurrence, or as direct evidence of a causal mechanism of recurrence. The observed gestational-age clustering should therefore be interpreted primarily as a descriptive clinical finding, rather than as evidence of an MVM-mediated biological mechanism. Future studies with standardized pathological assessment in both recurrent and first-time placental abruption cases, ideally with centralized and blinded review, are required to clarify whether MVM is associated with recurrence.

When clinical presentation and outcomes were compared, no significant differences were found between recurrent cases and multiparous first-time cases for many variables, including subjective symptoms, abnormal ultrasonographic findings, abnormal fetal heart rate tracing, blood loss, obstetric disseminated intravascular coagulation, blood transfusion, umbilical artery pH, intrauterine fetal death, and neonatal death. All recurrent cases were delivered by cesarean delivery, but the difference in cesarean delivery rate was not statistically significant. Birth weight was significantly higher in the recurrent group; however, gestational age at diagnosis and gestational age at delivery were only numerically higher and did not differ significantly. Given the very small recurrent group and the number of descriptive comparisons performed, this isolated finding may reflect the limited sample size or chance and should not be interpreted as evidence that recurrent cases were less severe or had better outcomes. Rather, the findings suggest that, aside from a history of placental abruption, the acute clinical presentation of recurrent cases largely overlapped with that of first-time cases in multiparous women. Given the limited number of recurrent cases, these negative findings should be interpreted cautiously. Specifically, the lack of statistically significant differences in these clinical and outcome variables should be regarded as inconclusive rather than as evidence that recurrent and first-time placental abruption have equivalent clinical severity or prognosis.

With respect to recurrence timing, the most important finding of this study was that recurrent episodes were centered around the gestational age at the previous episode. In clinical practice, there is often concern that recurrence may occur substantially earlier than the prior episode; however, in this cohort, most recurrent events occurred within ±2 weeks of the gestational age at the previous episode. At the same time, 3 of 11 recurrent events (27.3%) occurred outside this window. Two of these occurred 4 weeks later than the previous episode and recurred at term, whereas one occurred 5 weeks earlier, recurred at 34 weeks of gestation, and resulted in neonatal death in a patient who lived far from the hospital. Thus, while the gestational age at the previous episode may be a useful temporal reference for management in a subsequent pregnancy, it cannot fully account for all cases, and individualized care remains necessary with awareness that some recurrent cases may occur earlier than expected. This observation should not be interpreted as evidence that recurrence is unlikely outside the ±2-week window, and earlier planning may be necessary in selected situations, particularly when access to emergency obstetric care is limited.

Although the increased recurrence risk associated with a history of placental abruption has been consistently reported, high-level evidence remains limited regarding when to begin intensified surveillance, when inpatient management is appropriate, and when delivery intervention should be considered in a subsequent pregnancy [1,2,10,12,13]. Previous reports have suggested the need for special surveillance beginning well before the gestational age at the prior episode and have also raised the possibility of delivery intervention at around 37 weeks when the previous abruption occurred at term [1,2,10,12]. At the same time, available data remain insufficient to guide the choice between inpatient and outpatient management for chronic abruption and the optimal timing of delivery [1,13]. Importantly, the present findings reinforce the need for enhanced monitoring in pregnancies following a previous episode of placental abruption, even when conventional risk factors are absent.

Medical therapies aimed at improving uteroplacental circulation, such as low-dose aspirin, may theoretically be relevant in selected pregnancies complicated by ischemic placental disease-related conditions. In the present cohort, however, low-dose aspirin was used in only a small number of cases, including 1 of 10 women in the recurrent placental abruption group and 7 of 134 women in the first-time placental abruption group. Therefore, the present data cannot determine whether low-dose aspirin reduces the risk of recurrent placental abruption. Low-dose aspirin may be considered when there are established clinical indications, such as an increased risk of preeclampsia or other ischemic placental disease-related conditions; however, its use as a recurrence-prevention strategy for placental abruption cannot be supported by the present exploratory data.

Taken together, the present study does not establish a uniform management protocol, but it suggests the potential usefulness of using the gestational age at the previous episode as a practical temporal reference for planning individualized surveillance in a subsequent pregnancy [19]. In particular, the concept of progressively intensifying surveillance as the gestational age approaches that of the prior episode may be a feasible management option in tertiary care centers. Because approximately three-quarters of recurrent events in this study occurred within ±2 weeks of the previous episode, enhanced observation beginning before the gestational age of the previous episode, for example, approximately 2 weeks before in selected cases, may be considered as an individualized and exploratory approach. However, this 2-week threshold should be regarded as an exploratory guide derived from the present study rather than a universal criterion. Nevertheless, the present data cannot determine the optimal timing, frequency, or intensity of antenatal surveillance. In addition, practical use of the previous gestational age as a reference requires accurate information about the timing of the prior placental abruption, which may not always be available when previous obstetric records are incomplete or access to medical records is limited. In actual clinical practice, management should be individualized according to institutional resources, travel time to the hospital, severity of the previous episode, coexisting hypertensive disorders of pregnancy or fetal growth restriction, and patient behavior at symptom onset [1,2,20,21].

The fact that all recurrent cases in this cohort were delivered by cesarean delivery is a feature of this series, but it should not be generalized to suggest that the threshold for cesarean delivery should be lowered in women with a history of placental abruption. This discrepancy in mode of delivery may have influenced comparisons of some maternal and neonatal outcomes, including estimated blood loss and immediate neonatal status, and should be considered when interpreting the between-group comparisons. Actual delivery management depends on multiple factors, including fetal condition, the degree of suspicion of placental abruption, gestational age, cervical status, and institutional capacity, and should therefore be determined on an individual basis [2,20].

This study has several limitations. First, as a retrospective study based on medical record review, information bias cannot be fully excluded. In addition, because cases were collected over a long period across nine institutions, interinstitutional variation in diagnosis and management may have been present. Diagnostic ascertainment was based on institutional documentation and was not centrally adjudicated, which may have introduced misclassification bias.

Second, although gestational age was determined according to routine obstetric practice at each participating institution, information on the exact basis for gestational-age determination was not systematically collected. Therefore, some variation in the precision of gestational-age assessment may have affected the interpretation of the ±1-week and ±2-week clustering findings.

Third, recurrent placental abruption is rare, and the number of recurrent cases was limited. This restricted the ability to perform multivariable adjustment for confounding or detailed stratified analyses. More importantly, the study was not statistically powered to detect associations between recurrence and specific clinical characteristics, pathological findings, management factors, or maternal and neonatal outcomes. The absence of significant differences in many variables between recurrent and multiparous first-time cases may therefore reflect not only true similarity but also limited statistical power due to the small number of recurrent cases. Accordingly, non-significant findings should be interpreted as inconclusive and should not be taken as evidence that these factors are unrelated to recurrent placental abruption.

Fourth, the initiation of intensified surveillance and inpatient management was not based on a uniform protocol but on clinical judgment. Therefore, the present data cannot directly evaluate the effectiveness of specific interventions or determine the optimal timing, frequency, or intensity of antenatal surveillance, nor can they define the optimal timing of hospitalization or delivery.

Fifth, pathological evaluation was based mainly on original pathology reports and/or available archived slides from recurrent cases, and the availability and content of pathological data were not standardized across institutions or years. In particular, because MVM-related findings were not systematically documented in many historical pathology reports and archived slides from first-time placental abruption cases were not uniformly available, we could not perform a reliable pathological comparison between recurrent and first-time cases. In addition, centralized blinded pathological review was not performed, including within the recurrent cases. Therefore, diagnostic consistency of MVM-related findings could not be verified even among recurrent cases, and any apparent frequency of MVM-related findings may have been influenced by non-standardized pathological assessment, differences in reporting practices across institutions and years, and diagnostic heterogeneity. Accordingly, the present study could not evaluate whether MVM was associated with recurrent placental abruption.

Sixth, this study used a case–case descriptive design within a hospital-managed placental abruption cohort and was not designed to estimate recurrence risk in the general obstetric population or to identify independent predictors of recurrence. Because the total number of deliveries across the participating centers was not available as a denominator, institutional or regional incidence and recurrence rates could not be estimated. Selection bias related to the inclusion of hospital-managed cases may also have influenced the observed recurrence proportion and recurrence-timing distribution. Women with milder abruptions managed elsewhere, those who delivered at other hospitals, and those who experienced precipitous delivery before arrival at, or were otherwise not referred to, a participating tertiary/perinatal center may not have been captured and may have differed from the included cases. Accordingly, the observed recurrence-timing pattern may not be representative of the entire population of women with recurrent placental abruption. Therefore, negative findings and estimates based on the small recurrent group should be interpreted cautiously and should be considered hypothesis-generating rather than definitive evidence for or against specific associations.

## 5. Conclusions

In this multicenter retrospective descriptive study, recurrent placental abruption tended to occur within approximately ±1 to ±2 weeks of the gestational age of the previous episode, with 6 of 11 recurrent events occurring within ±1 week and 8 of 11 occurring within ±2 weeks of the previous gestational age. Because the recurrence analysis was based on only 11 events, the observed temporal clustering should be considered unstable, and both the timing pattern and the estimated proportions may change substantially in larger cohorts. These findings may assist clinicians when individualizing surveillance strategies but should not be interpreted as supporting a specific surveillance protocol. At the same time, recurrent cases did not show a clearly distinct clinical phenotype apart from their history, and neither gestational age at diagnosis nor gestational age at delivery differed significantly. However, because the number of recurrent cases was limited, the present study was underpowered to detect certain associations between recurrence and specific clinical characteristics, pathological findings, management factors, or maternal and neonatal outcomes. Therefore, these negative findings should be interpreted as inconclusive rather than as evidence of no association. Because pathological assessment was non-standardized and comparable pathological data from first-time cases were not systematically available, the present study also cannot determine whether maternal vascular malperfusion is associated with recurrence or represents a recurrence-specific pathological mechanism. The present data cannot determine the optimal timing, frequency, or intensity of antenatal surveillance or define the optimal timing of hospitalization or delivery. Surveillance should therefore be individualized in light of patient-specific factors, including delayed access to care among women living far from medical facilities. Because only hospital-managed cases were included, the observed recurrence-timing pattern may not be representative of the entire population of women with recurrent placental abruption. The findings should also be interpreted in the context of potential variation in gestational-age assessment, non-standardized pathological assessment, and the very small number of recurrent cases. Substantially larger multicenter studies are needed to confirm the timing estimates and clarify clinical, pathological, and management-related factors associated with recurrent placental abruption.

## Figures and Tables

**Table 1:** Baseline characteristics of the overall hospital-managed placental abruption cohort.

**1. Maternal characteristics**	
Maternal age, years	33.0 (19–45)
Advanced maternal age (≥35 years), *n* (%)	87 (36.4)
Pre-pregnancy body mass index, kg/m^2 a^	21.0 (15.0–38.1)
Smoking during pregnancy, *n* (%)	10 (4.2)
Conception by assisted reproductive technology, *n* (%)	9 (3.8)
Low-dose aspirin use, *n* (%)	8 (3.3)
**2. Parity**	
Nulliparous, *n* (%)	95 (39.7)
Multiparous, *n* (%)	144 (60.3)
**3. Preexisting medical conditions**	
Chronic hypertension, *n* (%)	3 (1.3)
Preexisting diabetes mellitus, *n* (%)	3 (1.3)
Thyroid disease, *n* (%)	6 (2.5)
Systemic autoimmune disease, *n* (%)	2 (0.8)
Renal disease, *n* (%)	2 (0.8)
Uterine leiomyomas or adenomyosis, *n* (%)	9 (3.8)
**4. Pregnancy characteristics in the index pregnancy**	
Hypertensive disorders of pregnancy, *n* (%)	44 (18.4)
Fetal growth restriction, *n* (%)	27 (11.3)
Multiple pregnancy, *n* (%)	2 (0.8)
Premature rupture of membranes, *n* (%)	21 (8.8)
Abdominal trauma during pregnancy, *n* (%)	4 (1.7)
**5. Recurrence status**	
Recurrent placental abruption, *n* (%)	10 (4.2)
First-time placental abruption in multiparous women, *n* (%)	134 (56.1)

Data are presented as *n* (%) or median (range). The observed recurrence proportion should not be interpreted as a population-level recurrence estimate because the study included only hospital-managed placental abruption cases and did not include all deliveries as a denominator. ART, assisted reproductive technology. ^a^ Pre-pregnancy BMI was available for 177/239 women. Thyroid disease included Graves’ disease (*n* = 1), Hashimoto’s disease (*n* = 1), hypothyroidism (*n* = 2), hyperthyroidism (*n* = 1), and thyroid cancer (*n* = 1). Systemic autoimmune disease included ulcerative colitis (*n* = 1) and autoimmune hepatitis (*n* = 1). Renal disease included IgA nephropathy (*n* = 2).

**Table 2:** Maternal characteristics and obstetric history of multiparous women with first-time versus recurrent placental abruption.

	Multiparous Women Without Previous Placental Abruption (*n* = 134)	Multiparous Women with Recurrent Placental Abruption (*n* = 10)	*p*-Value
**1. Maternal characteristics**			
Maternal age, years	34.0 (30.0–37.0)	35.5 (34.0–40.5)	0.039
Advanced maternal age (≥35 years), *n* (%)	55 (41.0)	5 (50.0)	0.742
Pre-pregnancy BMI, kg/m^2 a^	20.9 (19.3–24.1)	22.3 (21.1–23.2)	0.405
Smoking during pregnancy, *n* (%)	9 (6.7)	0 (0.0)	1.000
Conception by ART, *n* (%)	5 (3.7)	0 (0.0)	1.000
Low-dose aspirin use, *n* (%)	7 (5.2)	1 (10.0)	0.446
**2. Obstetric history**			
Parity, *n* (%)			0.130
1	82 (61.2)	4 (40.0)	
2	34 (25.4)	5 (50.0)	
3	14 (10.4)	0 (0.0)	
≥4	4 (3.0)	1 (10.0)	
Previous cesarean delivery, *n* (%)	19 (14.2)	1 (10.0)	1.000
Previous preterm birth, *n* (%)	12 (9.0)	1 (10.0)	1.000
Previous hypertensive disorders of pregnancy, *n* (%)	9 (6.7)	1 (10.0)	0.525
Previous fetal growth restriction, *n* (%)	3 (2.2)	1 (10.0)	0.253
**3. Preexisting medical conditions**			
Chronic hypertension, *n* (%)	3 (2.2)	0 (0.0)	1.000
Preexisting diabetes mellitus, *n* (%)	3 (2.2)	0 (0.0)	1.000
Uterine leiomyomas or adenomyosis, *n* (%)	7 (5.2)	0 (0.0)	1.000
**4. Pregnancy characteristics in the index pregnancy**			
Hypertensive disorders of pregnancy, *n* (%)	24 (17.9)	1 (10.0)	1.000
Fetal growth restriction, *n* (%)	17 (12.7)	1 (10.0)	1.000
Multiple pregnancy, *n* (%)	1 (0.7)	0 (0.0)	1.000
Premature rupture of membranes, *n* (%)	10 (7.5)	0 (0.0)	1.000
Abdominal trauma during pregnancy, *n* (%)	3 (2.2)	0 (0.0)	1.000

Data are presented as *n* (%) or median (IQR). Continuous variables were compared using the Mann–Whitney U test. Categorical variables were compared using Fisher’s exact test. Parity was compared using the Fisher–Freeman–Halton exact test. ^a^ Pre-pregnancy BMI was available for 96/134 women without previous placental abruption and 6/10 women with recurrent placental abruption. ART, assisted reproductive technology.

**Table 3:** Clinical presentation, pregnancy course, and maternal and neonatal outcomes among multiparous women with first-time versus recurrent placental abruption.

Clinical Variables and Outcomes	Multiparous Women Without Previous Placental Abruption (*n* = 134)	Multiparous Women with Recurrent Placental Abruption (*n* = 10)	*p*-Value
**1. Clinical presentation (symptoms)**			
Any subjective symptom, *n* (%)	121 (90.3)	10 (100.0)	0.600
Vaginal bleeding, *n* (%)	87 (64.9)	7 (70.0)	1.000
Abdominal pain, *n* (%)	37 (27.6)	5 (50.0)	0.156
Uterine contractions, *n* (%)	37 (27.6)	2 (20.0)	0.729
Leakage of fluid/suspected PROM, *n* (%)	11 (8.2)	1 (10.0)	0.593
Decreased fetal movement, *n* (%)	8 (6.0)	0 (0.0)	1.000
Back pain, *n* (%)	3 (2.2)	1 (10.0)	0.253
**2. Clinical findings at presentation**			
Any abnormal ultrasound finding, *n* (%)	89 (66.4)	4 (40.0)	0.166
Retroplacental hematoma, *n* (%)	59 (44.0)	2 (20.0)	0.191
Placental thickening, *n* (%)	26 (19.4)	0 (0.0)	0.209
Increased placental echogenicity or heterogeneous placental echotexture, *n* (%)	7 (5.2)	1 (10.0)	0.446
Sonographic evidence of placental separation, *n* (%)	8 (6.0)	0 (0.0)	1.000
Abnormal fetal heart rate tracing, *n* (%)	59 (44.0)	3 (30.0)	0.515
**3. Pregnancy course and delivery**			
Gestational age at diagnosis, weeks	34.0 (32.0–37.0)	36.0 (34.3–37.0)	0.172
Gestational age at delivery, weeks	35.0 (32.0–37.0)	36.5 (35.3–37.0)	0.092
Mode of delivery			
Spontaneous vaginal delivery, *n* (%)	21 (15.7)	0 (0.0)	0.358
Vacuum-assisted vaginal delivery, *n* (%)	3 (2.2)	0 (0.0)	1.000
Cesarean delivery, *n* (%)	110 (82.1)	10 (100.0)	0.214
**4. Maternal outcomes**			
Estimated blood loss, mL	890 (608–1328)	845 (706–934)	0.568
Obstetric disseminated intravascular coagulation, *n* (%)	20 (14.9)	0 (0.0)	0.358
Blood transfusion, *n* (%)	32 (23.9)	1 (10.0)	0.455
**5. Neonatal outcomes**			
Birth weight, g	2146 (1543–2560)	2600 (2287–2888)	0.011
Umbilical artery pH ^a^	7.266 (7.098–7.322)	7.330 (7.229–7.348)	0.192
Intrauterine fetal death, *n* (%)	19 (14.2)	0 (0.0)	0.359
Neonatal death, *n* (%)	2 (1.5)	1 (10.0)	0.195

Data are presented as *n* (%) or median (IQR). Categorical variables were compared using Fisher’s exact test. Continuous variables were compared using the Mann–Whitney U test. ^a^ Umbilical artery pH was available for 94/134 women without previous placental abruption and 7/10 women with recurrent placental abruption.

## Data Availability

The datasets generated and/or analyzed during the current study are not publicly available because they contain information that could compromise participant privacy. Data may be available from the corresponding author upon reasonable request and subject to approval by the participating institutions.

## Abbreviations

The following abbreviations are used in this manuscript:

ART	assisted reproductive technology
BMI	body mass index
CI	confidence interval
GA	gestational age
HDP	hypertensive disorders of pregnancy
IQR	interquartile range
MVM	maternal vascular malperfusion
PROM	premature rupture of membranes
